# Supplementary material for: Decoupling of Nrf2 Expression Promotes Mesenchymal State Maintenance in Non-Small Cell Lung Cancer
Source: Cancers (Basel). 2019 Oct 2;11(10):1488. doi: 10.3390/cancers11101488 (PMC6826656; doi:10.3390/cancers11101488)
Supplement: Supplementary file 1 [file cancers-11-01488-s001.pdf]

A

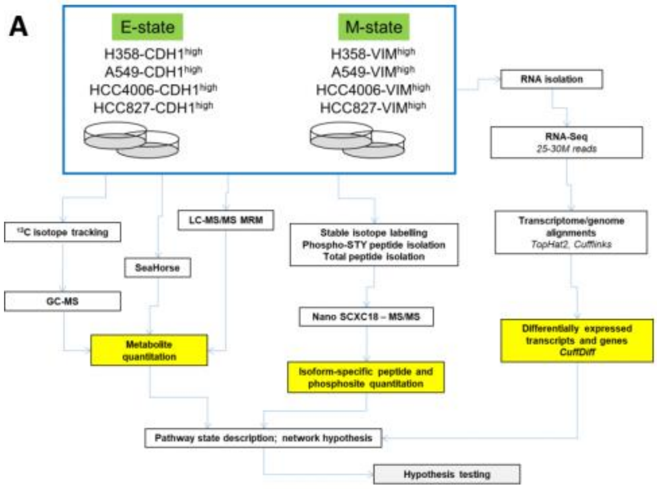

B

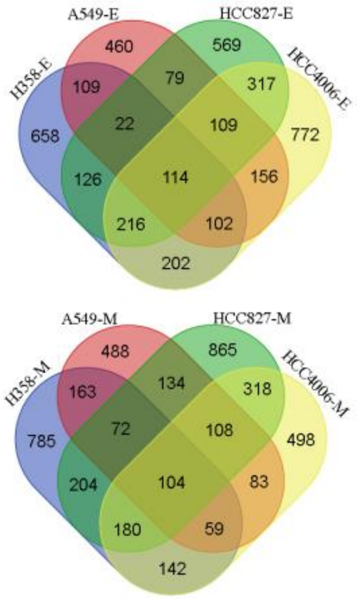

C

H358	A549	HCC4006	HCC827	
**KRAS G12C**; GRM1 R684L; RIMS2 S289W	**KRAS G12S**; GRM1 K697N; KEAP1 G333C	**EGFR ELR746del**; TP53 Y205H; NELL2 R425G;	**ELREA746del**; TP53 V218del; RIMS2 K1134fs	
1.58	0.98	1.08	3.33	FGF2
1.65	1.53	0.57	0.43	FOXO1
0.63	2.58	0.88	1.06	FOXP1
0.91	0.24	1.52	0.59	MYC
3.70	1.54	1.83	8.81	NOG
0.19	0.37	1.10	2.61	TUBB6
3.33	8.16	3.36	2.17	WNT5A
2.58	3.39	3.82	4.40	WNT5B
	-1.00	-3.77	-5.34	AQP5
-4.31	-0.92	-4.96	-2.72	FOXA2
-1.58	-3.46	-2.32	-0.98	FOXJ1
-5.22		-6.32	-1.01	HOPX
-1.28	-0.61	-1.05	-2.58	HOXA7
-3.08		-5.31	-1.00	NKX2-1

D

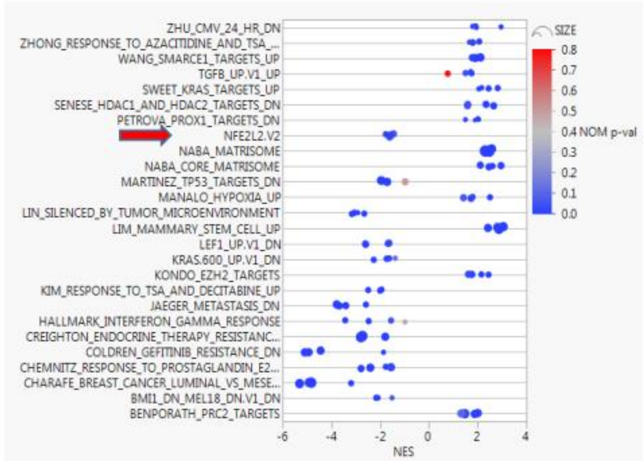

Figure S1. Isogenic NSCLC EMT models. **(A)**. Experimental schema **(B).** Molecular heterogeneity in M and E cell states measured by the number of genes with greater than two-fold change in RNA abundance (RNAseq) between M and E states. **(C).** RNA abundance characteristics of the four isogenic lung E and M state models (log2M/E fold change; bold values indicate FDR adjusted q value <0.05). **(D).** Comparison of E and M cell states for the four isogenic cell models by gene set enrichment (GSEA). Selected GSEA scores and p values correlating model RNA expression changes with selected signatures of NFE2L2/Nrf2 activation, hypoxia, epigenetic switching and expected EMT related signatures (Coldren, Creighton, TGFβ, LEF1). GSEA normalized enrichment score (NES), *p*-value (object color) and sample size (object size) are shown.

A

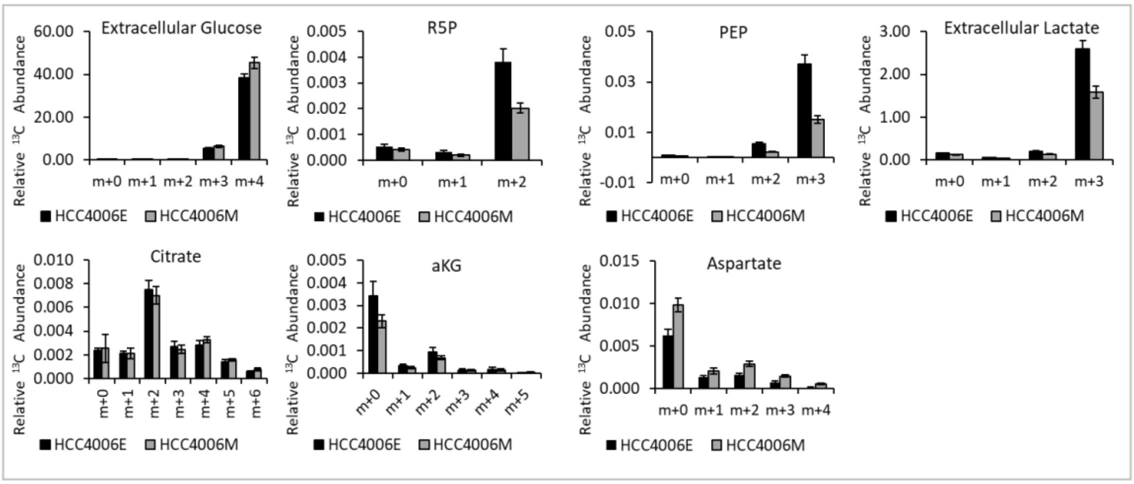

B

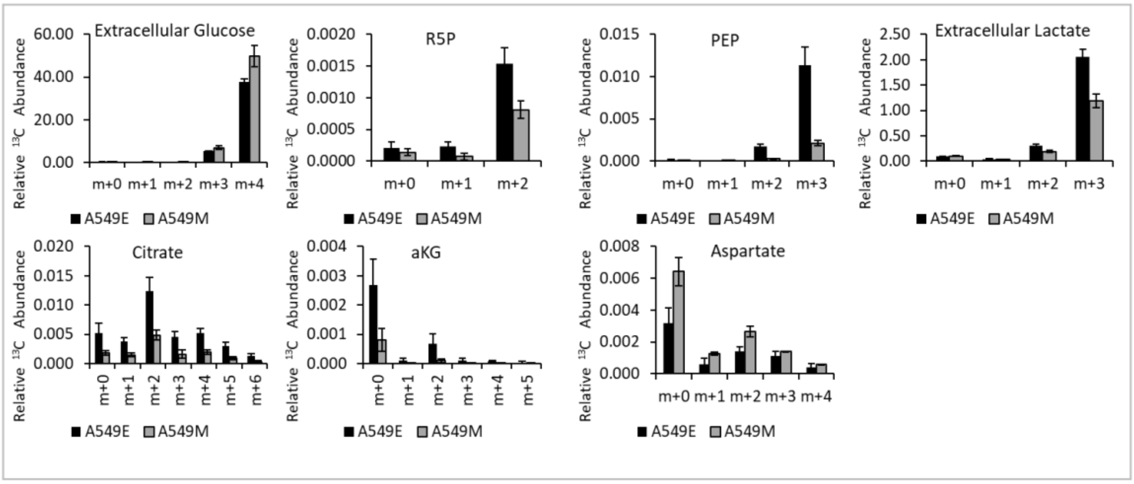

Figure S2. Isotopologue distributions of glycolytic and TCA cycle metabolites in epithelial and mesenchymal cell states. (**A**). HCC4006 treated with 13C6-glucose; glycolytic, PPP, TCA cycle and glucose isotopologue distributions (Figure 2). (**B**). A549 treated with 13C6-glucose; glycolytic, PPP, TCA cycle and glucose isotopologue distributions (Figure 2).

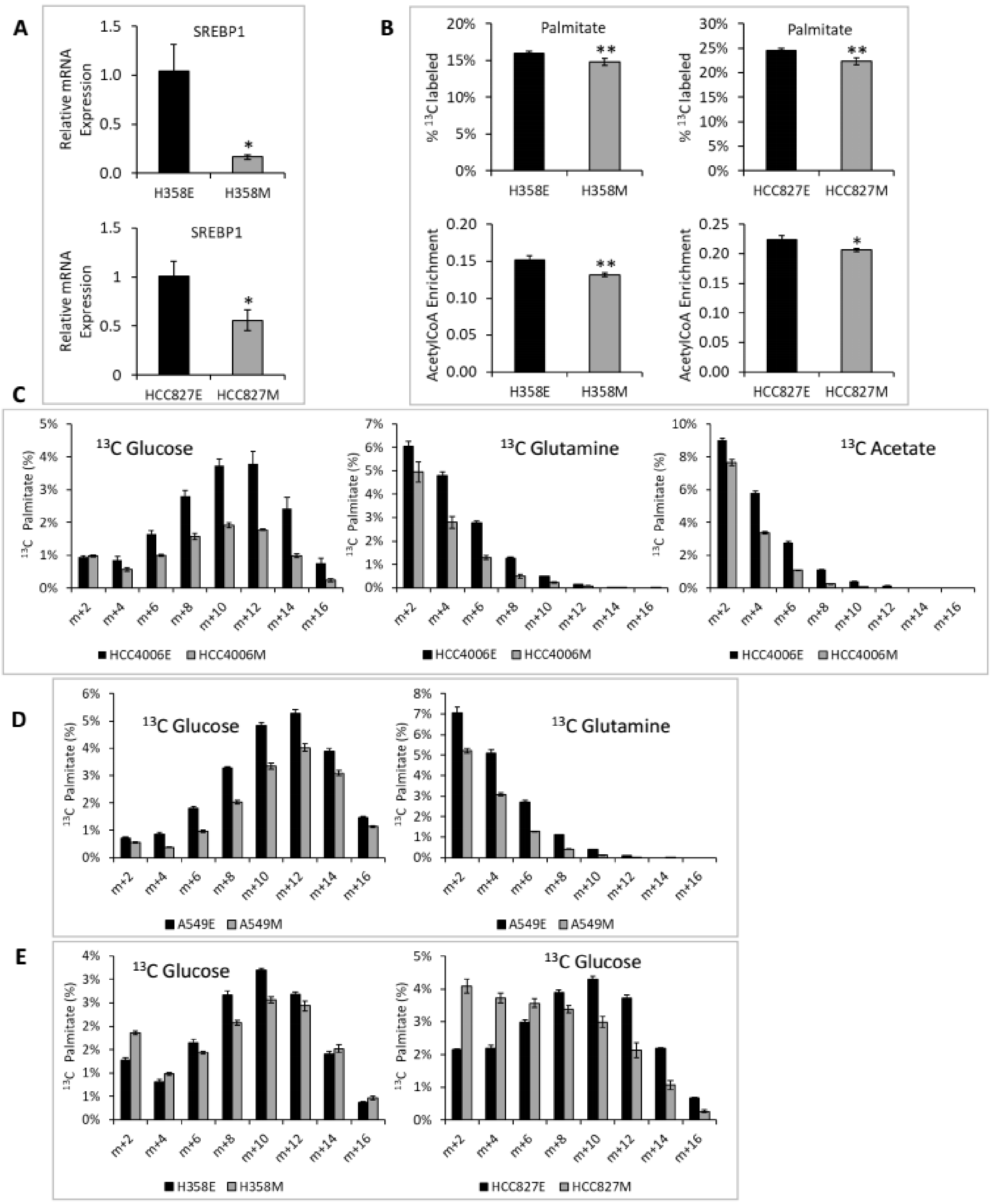

Figure S3. Isotopologue distributions of *de novo* lipid synthesis metabolites in epithelial and mesenchymal cell states. (**A**). H358 and HCC827 SREBP1/SREBF1 RNA is reduced in the M state. (**B**). Similar to A549 and HCC4006 cells (Figure 3), H358 and HCC827 show decreased incorporation of 13C6-glucose into palmitate as well as decreased acetyl-CoA enrichment in the M state by GC-MS. (**C**). HCC4006 cells show decreased incorporation of 13C6-glucose, 13C5-glutamine and 13C2-acetate into palmitate in the M state. (**D**). A549 cells show decreased incorporation of 13C6-glucose and glutamine into palmitate in the M state. (**E**). H358 and HCC827 cells show decreased incorporation of 13C6-glucose into palmitate in the M state.

A

Total Metabolite LC-MS/MS	HCC4006 log2 FC	p-value	A549 log2 FC	p-value
Ornithine	0.14	0.090	0.11	0.013
**Carbamoyl phosphate**	1.81	0.000	3.68	0.000
Citrulline	0.02	0.769	-0.25	0.001
**L-Aspartate**	1.01	0.017	0.20	0.000
L-Arginino-Succinate	0.51	0.018	-0.57	0.112
L-Arginine	0.19	0.039	-0.06	0.277
Urea	-0.24	0.099	0.49	0.223
L-Glutamate	0.26	0.293	0.00	0.996

B

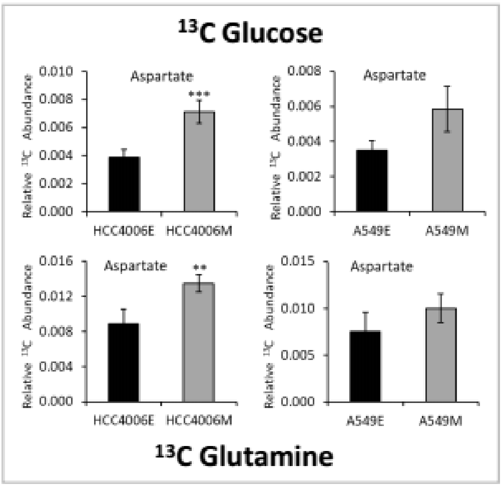

C

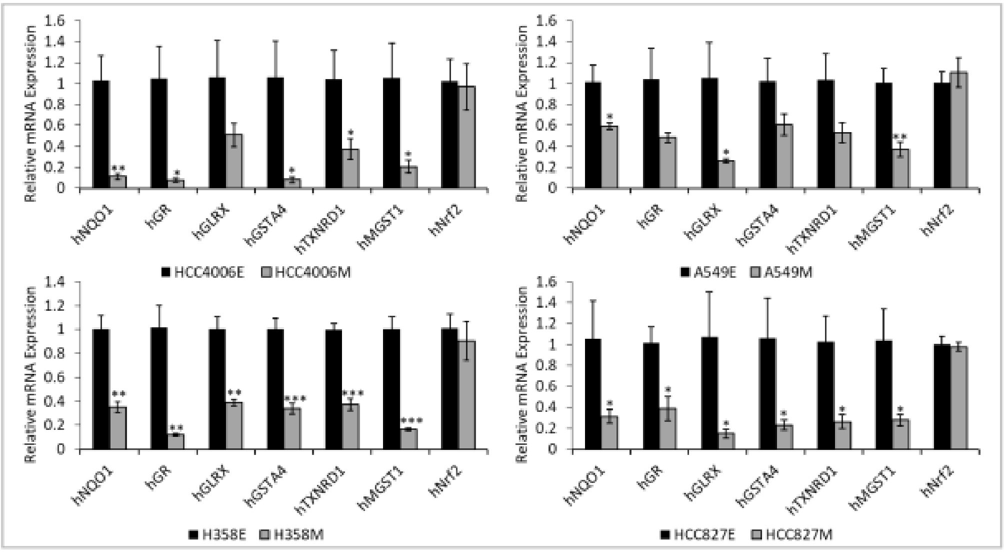

D

H358	HCC827	HCC4006	A549	
-0.68	-0.77	-0.44	-0.10	CBR1
-0.73	-0.38	-1.79	-1.47	GCLC
-0.31	-0.65	-2.20	-1.41	GLRX
-2.99	-2.23		-9.21	GPX2
-1.96	-0.79	-0.75	0.49	GSR
-1.29	-1.79	-5.79	-3.53	GSTA4
-2.32	-0.17	-1.00	-1.54	MGST1
-2.67	-0.21	-1.20	-0.92	NQO1
-0.62	-0.93	-0.62	-0.01	PRDX5

E

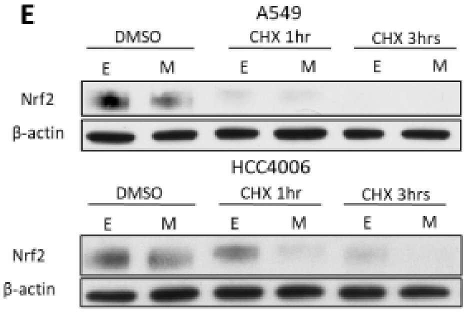

Figure S4. Altered metabolic and redox state RNAs and protein between EMT states. (**A**). Urea cycle metabolites measured by LC-MS/MS in HCC4006 and A549 M states; both show increases in carbamoyl phosphate and aspartate while other urea cycle metabolites show little to no consistent changes. (**B**). Accumulation of aspartate in Mstate measured by 13C6-glucose and 13C5-glutamine incorporation ($p < 0.05$ *; $p < 0.01$ **; $p < 0.001$ ***). (**C**). Nrf2 target RNAs decrease in the M state as measured by RT-PCR, but Nrf2 RNA levels remain unchanged ($p < 0.05$ *; $p < 0.01$ **; $p < 0.001$ ***). **(D)** Nrf2 target RNAs decrease in the mesenchymal state (RNAseq log2 M/E). **(E)** A549 and HCC4006 were treated with CHX for 1 and 3 hours to determine stability of existing Nrf2.

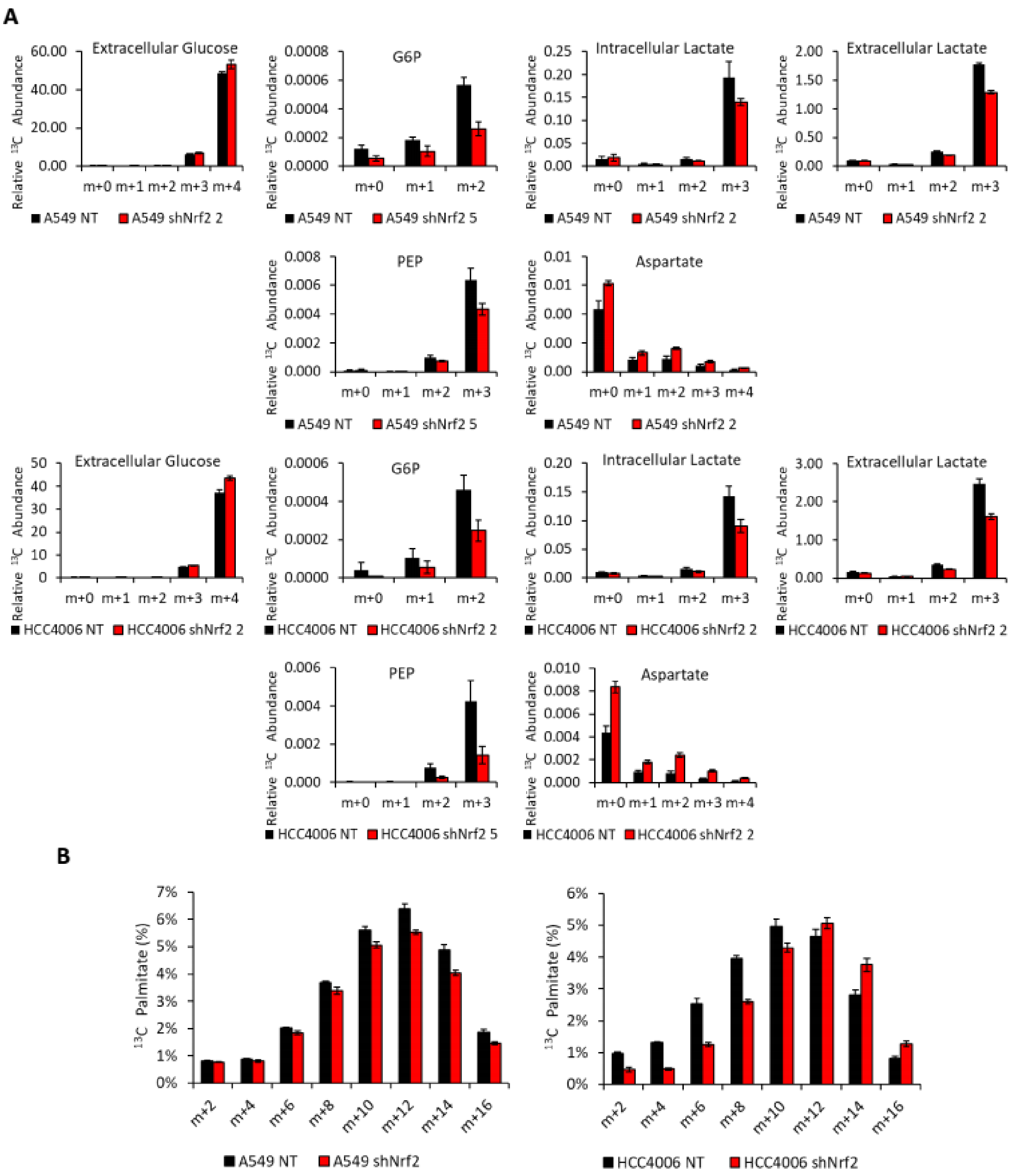

Figure S5. Isotopologue distributions of palmitate, glycolytic and TCA cycle metabolites in epithelial and mesenchymal cell states. (**A**). Isotopologue distributions for glucose, G6P, lactate, PEP, and aspartate in A549 and HCC4006 shNrf2 cells (Figure 6). (**B**). Isotopologue distributions for palmitate in A549 and HCC4006 shNrf2 cells (Figure 6).

A.

Gene	ΔNrf2 inactive - active [n=503]	q-Value [n=503]	Co-correlation CDH1	Co-correlation VIM
KRTCAP3	-1.03	0.000	0.71	-0.59
RAB25	-0.54	0.000	0.80	-0.50
TMEM45B	-0.49	0.001	0.53	-0.41
BSPRY	-0.48	0.000	0.75	-0.44
CLDN7	-0.45	0.000	0.80	-0.51
ELMO3	-0.40	0.000	0.71	-0.52
TJP3	-0.37	0.006	0.75	-0.61
CRB3	-0.33	0.001	0.69	-0.43
MPP7	-0.28	0.014	0.65	-0.44
GRHL2	-0.28	0.001	0.74	-0.59
AP1M2	-0.26	0.001	0.70	-0.45
MAL2	-0.25	0.031	0.71	-0.50
STAP2	-0.24	0.013	0.70	-0.49
SPINT2	-0.24	0.004	0.73	-0.44
TMC4	-0.24	0.019	0.77	-0.56
SH3YL1	-0.22	0.019	0.68	-0.56
PRR5	-0.22	0.025	0.76	-0.48
TNFRSF21	0.26	0.038	0.48	-0.22
SHROOM3	0.26	0.042	0.67	-0.36
NRP1	0.49	0.000	-0.01	0.15
TGFBI	0.52	0.003	0.07	0.09
ZEB1	0.55	0.000	-0.78	0.62
VIM	0.57	0.000	-0.55	1.00
ANTXR2	0.60	0.000	-0.26	0.40
LIX1L	0.66	0.000	-0.67	0.54
FN1	0.66	0.000	0.15	0.27
AXL	0.70	0.000	-0.45	0.60
MMP2	0.76	0.000	-0.27	0.30
GALNT5	0.98	0.000	0.15	0.22
CDH3	1.14	0.000	0.75	-0.42

B.

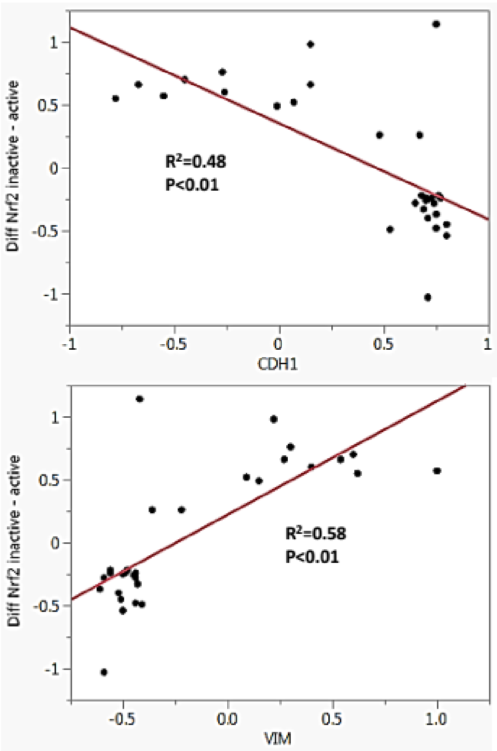

Figure S6. Attenuation of Nrf2-dependent transcription correlates with the mesenchymal RNA expression in NSCLC adenocarcinomas. (**A**). Co-correlation of Nrf2 target RNA expression (anchor genes NQO1, GSR, GPX2, GSTA4, PRDX5, CBR1, GLRX, MGST1, GCLC) with a reference 85 gene EMT signature defined [1]. The TCGA Provisional 503 patient lung adenocarcinoma patient dataset was queried. Nrf2 target RNA expression was positively correlated with an epithelial state. Nrf2 activation correctly correlated with KEAP1 mutation (q = 2.4 × 10^{-4}; not shown). (**B**). Regression analysis of co-correlated Nrf2 genes and EMT signature genes detected.

A

Gene	ΔNrf2 inactive - active [n=203]	q-Value [n=203]	Co-correlation CDH1	Co-correlation VIM
KRTCAP3	-1.52	0.000	0.71	-0.59
CLDN7	-0.76	0.000	0.80	-0.51
RAB25	-0.71	0.000	0.80	-0.50
ELMO3	-0.65	0.000	0.71	-0.52
CRB3	-0.63	0.000	0.69	-0.43
BSPRY	-0.53	0.001	0.75	-0.44
TMEM125	-0.53	0.023	0.78	-0.55
STAP2	-0.45	0.006	0.70	-0.49
TJP3	-0.45	0.021	0.75	-0.61
PRR5	-0.43	0.011	0.76	-0.48
AP1M2	-0.30	0.021	0.70	-0.45
SPINT2	-0.28	0.033	0.73	-0.44
TNFRSF21	0.52	0.020	0.48	-0.22
VIM	0.55	0.002	-0.55	1.00
NRP1	0.57	0.004	-0.01	0.15
ZEB1	0.60	0.000	-0.78	0.62
TGFBI	0.60	0.045	0.07	0.09
LIX1L	0.64	0.000	-0.67	0.54
ANTXR2	0.71	0.000	-0.26	0.40
AXL	0.78	0.000	-0.45	0.60
FN1	0.85	0.003	0.15	0.27
MMP2	0.88	0.000	-0.27	0.30
DSP	0.89	0.011	0.70	-0.56
CDH3	1.29	0.003	0.75	-0.42
GALNT5	1.33	0.000	0.15	0.22

B

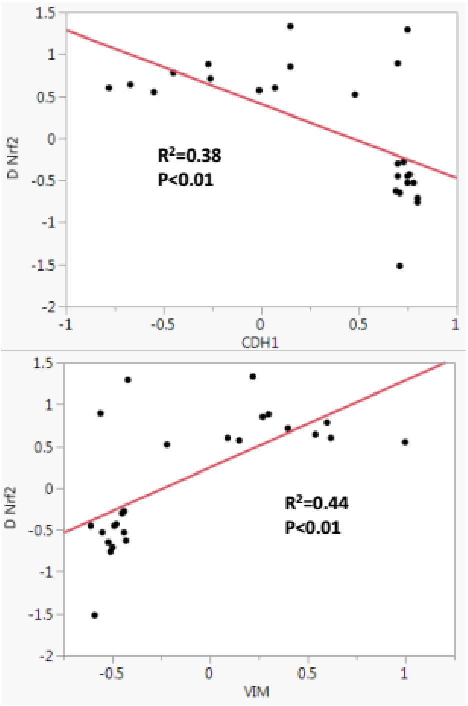

Figure S7. Attenuation of Nrf2-dependent transcription correlates with the mesenchymal-like tumor cell state in NSCLC adenocarcinomas. (**A**). Co-correlation of Nrf2 target RNA expression (anchor genes NQO1, GSR, GPX2, GSTA4, PRDX5, CBR1, GLRX, MGST1, GCLC) in 203 patient NSCLC adenocarcinoma TCGA patient dataset, with a reference 85 gene EMT signature [1]. (**B**). Regression analysis of co-correlated Nrf2 genes and EMT signature genes detected.

	Adenocarcinoma N=503		Adenocarcinoma N=203	
**Gene**	**Δ M-sig.**	**q-Value**	**Δ M-sig.**	**q-Value**
**Metabolic**				
AGR2	-1.11	0.0007	-1.28	0.0173
ASS1	-0.92	0.0001	-1.10	0.0180
BCAT1	0.83	0.0000	0.83	0.0136
BCAT2	-0.60	0.0000	-0.67	0.0001
CBR1	-0.62	0.0001	-0.74	0.0005
FA2H	-1.12	0.0002	-0.86	0.0425
FAAH	-1.09	0.0000	-1.03	0.0034
FAAH2	-0.92	0.0000	-1.07	0.0145
GPX2	-2.41	0.0001	-2.22	0.0176
GSTA4	-0.61	0.0002	-0.80	0.0042
HIF1A	0.78	0.0000	0.76	0.0005
LRP5	-0.38	0.0031	-0.23	0.2230
PFKFB2	-0.60	0.0000	-0.59	0.0017
PGK1	0.52	0.0000	0.34	0.0574
PRDX5	-0.34	0.0006	-0.44	0.0096
SREBF1	-0.48	0.0000	-0.42	0.0201
SREBF2	-0.29	0.0027	-0.22	0.1140
SYVN1	-0.36	0.0000	-0.24	0.0491
**Lung Development**				
FGF2	0.98	0.0000	0.72	0.0626
FOXA1	-0.79	0.0001	-0.62	0.0651
FOXJ1	-0.88	0.0077	-0.79	0.2010
FOXO1	0.25	0.0516	0.01	0.9540
FZD5	-0.50	0.0007	-0.48	0.0691
HBEGF	0.56	0.0008	0.55	0.0555
HOPX	-1.00	0.0008	-1.77	0.0003
MYC	0.37	0.0512	0.36	0.3310
NKX2-1	-1.52	0.0004	-1.83	0.0210
TUBB6	1.04	0.0000	1.02	0.0004
WNT5B	0.64	0.0039	0.75	0.0563

Figure S8. Co-correlation of EMT, metabolic and lung development RNA expression in lung adenocarcinoma tissue. Co-correlation of isogenic H358, A549, HCC827 and HCC4006 model EMT marker genes with metabolic and lung development RNA expression comparing NSCLC adenocarcinoma TCGA 503 (Provisional) and 203 (PMID: 25079552) patient datasets. Co-correlation anchor genes were FN1, VIM, LOX, GPC6 and ZEB1 (ΔM-sig). Mean differences between anchor gene correlations with TCGA RNA expression values are shown with FDR adjusted p-values (q-value) for the two patient sets.

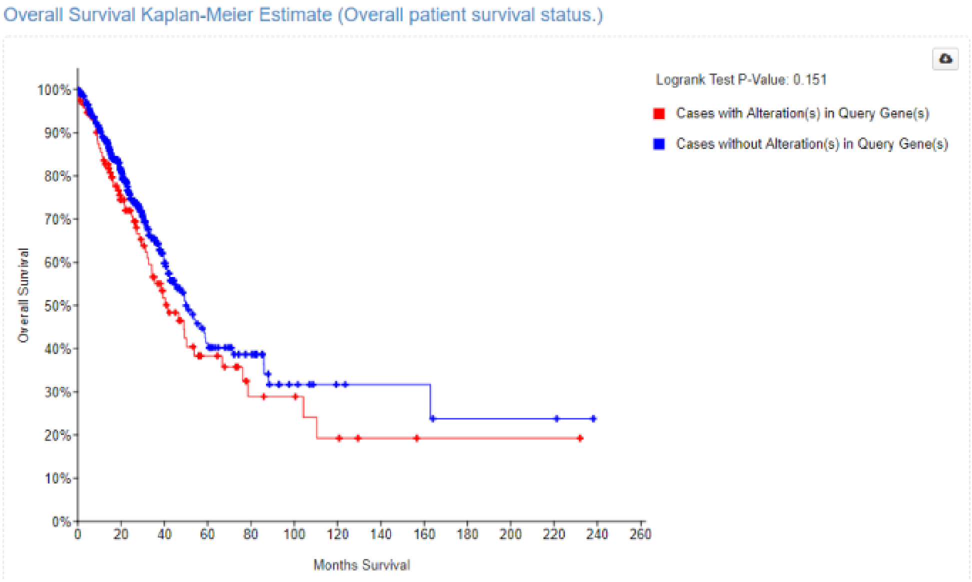

Figure S9. A trend for reduced overall survival in patients with high Nrf2 signature expression (p = 0.15) using a TCGA Provisional lung adenocarcinoma 586 sample set is observed. Future studies should include multivariate analysis of retrospective and prospective NSCLC adenocarcinoma patient datasets.

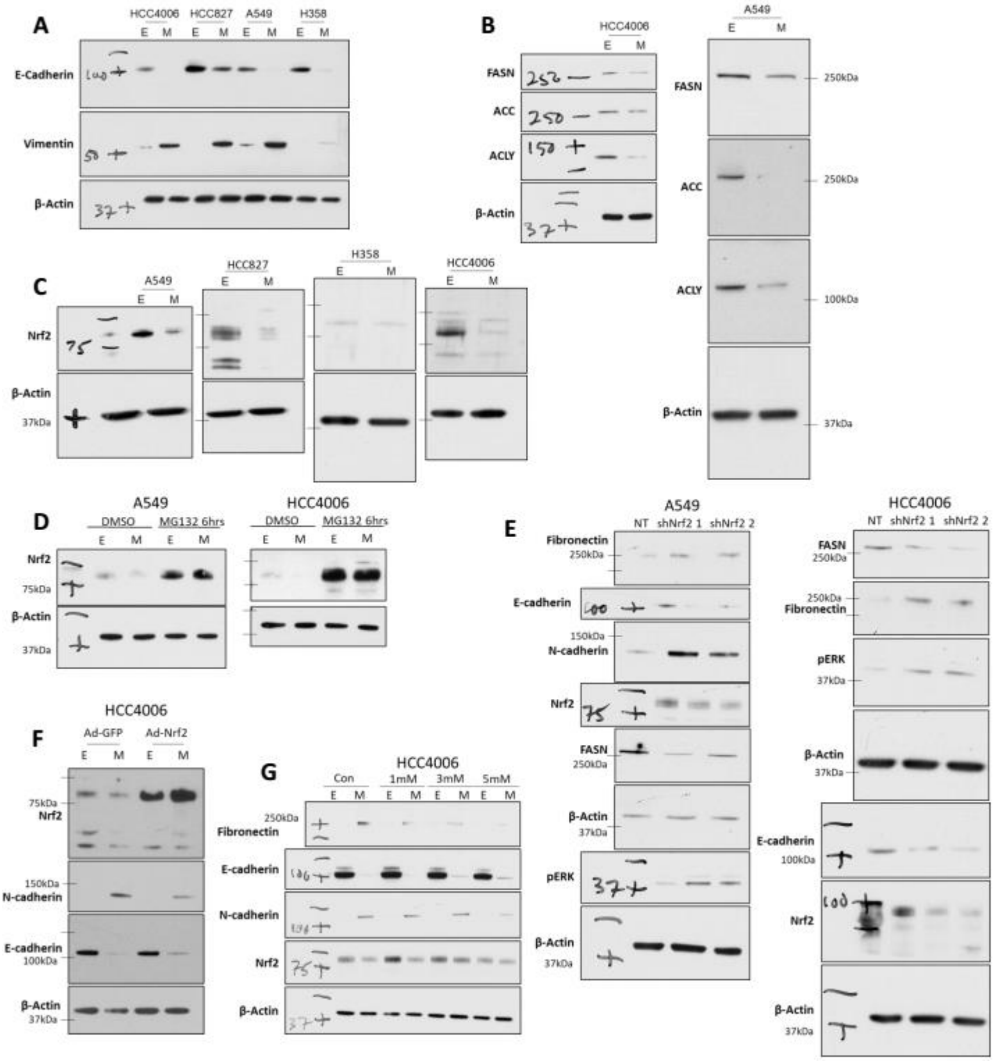

Figure S10. Full immunoblot scans with molecular weight markers. **(A).** Full blot scans for EMT state markers in all four cell lines (Figure 1B). **(B).** Full blot scans for lipogenic genes in A549 and HCC4006 E and M cell states (Figure 3A). **(C).** Full blot scans for Nrf2 protein levels in E and M cell states in all four cells lines (Figure 4D). **(D).** Full blot scans for MG132 proteasomal inhibition in both A549 and HCC4006 cell lines (Figure 4F). **(E).** Full blot scans for Nrf2 shRNA knockdown in both A549 and HCC4006 cell lines (Figure 5A). **(F).** Full blot scans for HCC4006 cells infected with adenovirus expressing GFP or Nrf2 (Figure 5D). **(G).** Full blot scans for HCC4006 cells treated with N-acetylcysteine (NAC) in both E and M cell states (Figure 5E).

Reference

1. Byers, L.A., et al., An epithelial-mesenchymal transition gene signature predicts resistance to EGFR and PI3K inhibitors and identifies Axl as a therapeutic target for overcoming EGFR inhibitor resistance. *Clin. Cancer Res*. **2013**, *19*, 279–290.
